# Brief Communication: The Predictable Network Topology of Evolutionary Genomic Constraint

**DOI:** 10.1093/molbev/msae033

**Published:** 2024-02-16

**Authors:** Katharina C Wollenberg Valero

**Affiliations:** School of Biology and Environmental Science, University College Dublin, Belfield, Ireland

**Keywords:** constraint, networks, evolution, pleiotropy, cost of complexity

## Abstract

Large-scale comparative genomics studies offer valuable resources for understanding both functional and evolutionary rate constraints. It is suggested that constraint aligns with the topology of genomic networks, increasing toward the center, with intermediate nodes combining relaxed constraint with higher contributions to the phenotype due to pleiotropy. However, this pattern has yet to be demonstrated in vertebrates. This study shows that constraint intensifies toward the network's center in placental mammals. Genes with rate changes associated with emergence of hibernation cluster mostly toward intermediate positions, with higher constraint in faster-evolving genes, which is indicative of a “sweet spot” for adaptation. If this trend holds universally, network node metrics could predict high-constraint regions even in clades lacking empirical constraint data.

## Introduction

In a genome, near-infinite numbers of gene modifications and resulting allelic combinations can emerge through mutation and natural selection. Phenotypic convergence and the reuse or reevolution of genes for similar adaptations raise the question of what genomic characteristics enable such seemingly improbable evolutionary outcomes (Futuyma 2010). Several studies have provided transcript-level, base-level, or genome-wide scores of constraint for various organisms, including *Anolis* lizards (Tollis et al. 2018), lacertid lizards (as tree root-to-tip-length, (Garcia-Porta et al. 2019)), whales (Tollis et al. 2019), birds (Zhang et al. 2014; Feng et al. 2020), and humans (Chen et al. 2023). Some genomic regions appear to retain mutations less frequently, leading to the formation of, for example, ultraconserved elements (Katzman et al. 2007; Faircloth et al. 2012), which were conserved to more than 98% across 240 placental mammal genomes (Christmas et al. 2023). Other genomic regions showed accelerated evolution crucial for lineage-specific adaptations across placental mammals (Christmas et al. 2023). In addition, both highly and lowly constrained regions across genomes appear to be homologous between mammals and birds, with ∼13% found within protein-coding sequences (Zhang et al. 2014). What remains unexplored in these studies is to which extent such elements of constraint arise through functional interactions among gene products, which have been proposed to limit the degrees of freedom for selection (Payne and Wagner 2019). Empirical classification of evolutionary constraint at the level of protein–protein interactions (PPI) networks is limited, particularly due to the scarcity of multiclade genomic alignments, especially in multicellular organisms (but see studies on yeast: Frost et al. 2012; Schoenrock et al. 2017; Wollenberg Valero 2020). The 240-mammal alignment within the Zoonomia genomic resource now enables a first test for the influence of network topology in shaping levels of evolutionary rate constraint and the location of nodes relative for mammal-specific adaptations (Christmas et al. 2023).

The recent publication by the Zoonomia consortium (Christmas et al. 2023) presented a comprehensive assessment of mammalian evolutionary rate constraint at single-base resolution using the “Phylop” metric. This metric (Pollard et al. 2010; Hubisz et al. 2011) describes the deviation of nucleotide substitution rates from neutrality in a clade-specific manner and allows discriminating between rate acceleration and deceleration. Significant Phylop scores [-log10(*P*-value)] different from 0 (neutral evolution) indicate higher levels of sequence constraint (positive) or acceleration (negative), with constraint reflecting functional importance maintained by purifying selection. Remarkably, the study found that around 10% of mammalian genomes exhibited strong constraint, often within crucial developmental pathways. Notably, while ∼80% of highly constrained regions occur outside of protein-coding exons, species-specific adaptations were observed in genes with relaxed constraint, such as a correlation between olfactory gene number and olfactory turbinals, enhancing environmental sensing capabilities (Christmas et al. 2023). Additionally, the study revealed that single-base constraint coincided with higher-level functional elements, including CTCF transcription factor binding sites and functionally important peptide regions like start/stop codons and splice sites (Christmas et al. 2023). Such constraints collectively ensure the functionality of cellular and organismal processes, extending from peptide functionality to genomic network constraint (Wollenberg Valero 2020).

In a previous study using yeast as an example, I demonstrated a role of network topology in determining constraint levels. Three network statistical parameters—average shortest path length (ASPL), betweenness centrality (BC), and neighborhood connectivity (NC)—can be utilized to identify adaptable versus resilient regions within the PPI network, contributing to our understanding of constraint dynamics (Wollenberg Valero 2020). The highest levels of constraint are expected in the central (hub) nodes of the network, characterized by the highest BC. Moving toward the network periphery, rate constraint gradually diminishes while passing through intermediate nodes with the highest NC, indicating a greater number of connections to other nodes. Finally, the peripheral nodes, marked by the highest ASPL and the fewest connections to other nodes, demonstrate the lowest levels of constraint (Wollenberg Valero 2020).

Here, I present a visualization and analysis of the PPI network topological architecture of mammalian genomic constraint, testing 4 hypotheses which are (i) that constraint is predicted by the structure of the network, (ii) that constraint differs between network node categories, (iii) that hibernation-associated genes are located in intermediate nodes in the network and differ in constraint values, and lastly (iv) that the positions of hibernation-relevant genes in the network are not just an outcome of chance.

## Results and Discussion

**Fig. 1.**
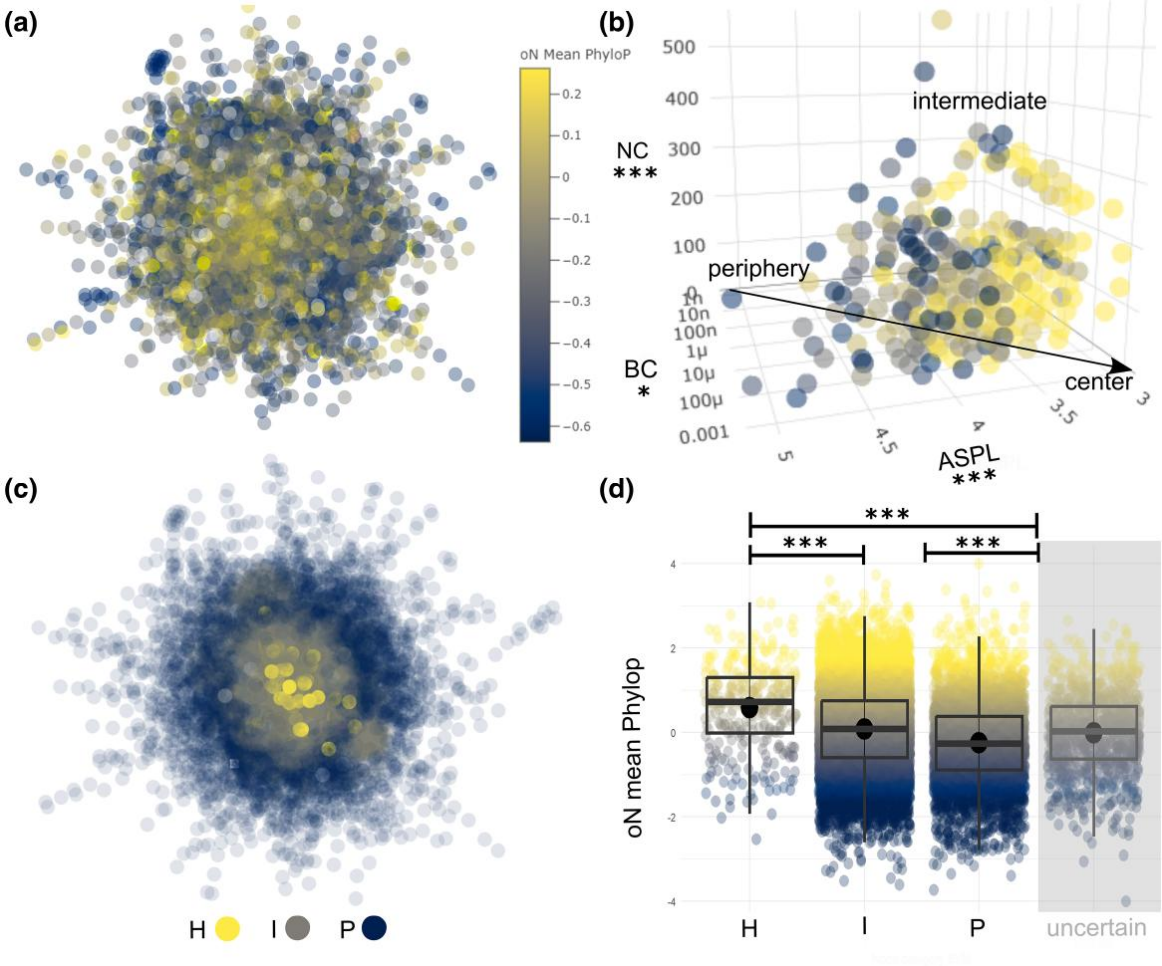
Visualization of the network topology of mammalian evolutionary constraint. a) The constraint metric mean Phylop computed by Christmas et al. (Christmas et al. 2023) is visualized on a high-confidence STRING human protein–protein interactome, showing constraint being higher in the center of the network. b) Reduced dimensions of mean Phylop plotted for 3 network statistic metrics ASPL, BC, and NC, representing node positions within network topology. These dimensions characterize the center, intermediate, and peripheral regions of the network. c) Network with nodes classified into categories H (yellow/bright), I (gray), and *P* (blue/dark) using ASPL, BC, and NC. d) Mean Phylop significantly changes with network node category, with constraint decreasing from the center to the periphery of the network. Significance levels for ANOVA (b) and Emmeans post hoc tests (d) are indicated with stars: ***P < 0.001, **P < 0.01, *P < 0.05.

In mammal genomes (modeled on a high-confidence STRING PPI network of *Homo sapiens*; Fig. 1), ASPL, NC, and BC emerge as significant predictors of base-level constraint summarized at gene level (*F* = 449.6, *P* < 0.0001, df = 15,780, explaining 8.55% of overall variance; supplementary table S1, Supplementary Material online), which divide protein-coding genes (nodes) within the network into 3 regions (center, intermediate, and periphery). Consistent with expectations, mammalian evolutionary constraint exhibits higher values in central hub nodes of the network, gradually declining toward the network periphery with significant pairwise differences in mean Phylop across 3 node categories—H (hub), I (intermediate), and *P* (peripheral; with a small but very strongly supported effect for the full model *F* = 218.6, *P* < 2e^−16^, df = 14,825, supplementary table S2, Supplementary Material online, and large to huge pairwise differences, supplementary table S3, Supplementary Material online). This pattern mirrors the findings previously observed in yeast evolutionary constraint (Wollenberg Valero 2020).

**Fig. 2.**
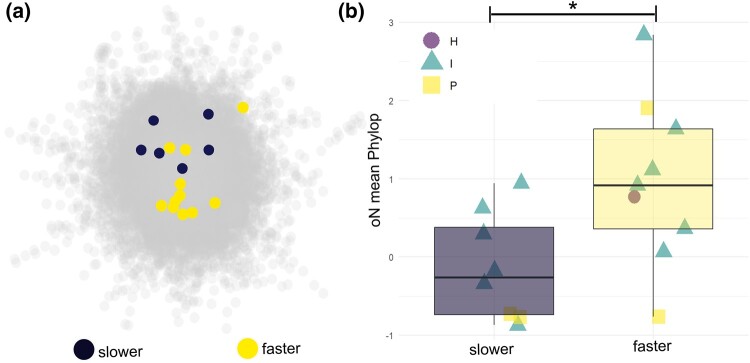
Association of constraint and network position in genes associated with hibernation phenotype. a) Genes having significantly accelerated (yellow/bright) or decelerated (blue/dark) evolutionary rates in association with the evolution of the hibernating phenotype from Christmas et al. (Christmas et al. 2023), within the human high-confidence STRING PPI. b) Association between the direction of evolution of hibernation-associated genes, their node class (purple circle, H; teal triangle, I; and yellow square, *P*), and rate constraint in mammals (orderNormalized mean Phylop). Boxes show medians and quartiles. Significance levels for ANOVA are indicated with stars: ***P < 0.001, **P < 0.01, *P < 0.05.

As predicted, only one of the genes whose evolutionary rate was significantly associated with hibernating phenotype in mammals was situated in hub nodes; instead, most were associated with intermediate nodes with only 4 out of 18 peripheral node positions (Fig. 2). Surprisingly, faster-evolving genes exhibited higher rate constraint compared with slower-evolving genes with large effect size and moderate statistical support (*F* = 6.229, *P* = 0.027; supplementary tables S4–S6, Supplementary Material online) and significant pairwise differences of small to medium effect size. This at first glance counterintuitive observation can be explained by the “cost of complexity” hypothesis (Wagner et al. 2008; Wang et al. 2010). Pleiotropic genes involved in multiple biochemical pathways and having numerous interaction partners are subject to evolutionary rate constraint due to purifying selection (Promislow 2004; He and Zhang 2006; Pavlicev and Wagner 2012), particularly in highly constrained hub nodes. Hibernation genes located in intermediate nodes showed higher constraint than those in peripheral nodes, although this trend did not reach statistical significance (supplementary table S5, Supplementary Material online). However, in general, genes with the highest number of connections are represented by intermediate node positions within the network, not by hub node positions (Wollenberg Valero 2020; Fig. 1). The combination of relatively lower constraint and higher complexity in nodes intermediate in the PPI can result in higher rates of accumulation of mutations. These mutations, if beneficial, can in turn have a greater phenotypic effect compared with nodes at the periphery, which accumulate adaptations with least constraint but also with lower phenotypic effects due to a lower degree of pleiotropic interactions (Wollenberg Valero 2020). This pattern is mirrored by genes decelerating in response to hibernation being under lower constraint. Consequently, nodes with intermediate position and rate constraint are good candidates for the emergence of novel and rapid phenotypic adaptations. Additional support for the intermediate network of hibernation-relevant genes not being just an outcome of chance comes from the fact that their ASPL, NC, BC, and Phylop values were significantly different from 1,000 random draws (supplementary tables S7 to S10, Supplementary Material online).

Using tools like STRING V12.0, one can now easily obtain networks by uploading user-generated genomes or accessing over 10,000 existing organismal networks (Szklarczyk et al. 2023). Meanwhile, inferring base-level constraint still requires phylogenetic sampling and analysis of related genomes. The observed correlation between constraint and network position in model organisms such as yeast and humans indicates that network data might adequately predict constraint. Thus, networks could serve as proxies to identify genome regions with lower constraint and high complexity—those both able to retain mutations accessible to selection and capable of phenotypic change—therefore allowing to test hypotheses about the evolvability of specific genes or gene clusters.

## Conclusion

The predictability of node constraint based on network topology previously shown for yeast and demonstrated here for mammals could be a universal property of genomic networks, which opens up the possibility of using network properties as a predictive tool for identifying genomic regions with high constraint and potential for adaptation. This potentially has wide-ranging applications, including the prediction of disease heritability, phenotypic evolutionary constraint (Christmas et al. 2023), and even the identification of genomic regions where organisms may or may not respond to rapid climate change (Wollenberg Valero et al. 2021). Such predictive capabilities would be particularly valuable for animal groups or scenarios where empirical classification of evolutionary genomic constraint values is still lacking.

## Methods

A human protein–protein interactome was obtained via NDex from the STRING database and loaded into Cytoscape (V3.10). This “high-confidence” network contained 17,185 nodes and 420,534 edges and was limited to edges with confidence scores > 0.7 to exclude edges with spurious associations (V12.0) (Szklarczyk et al. 2017; Szklarczyk et al. 2023). The median confidence score of this network was 0.903, which is a summary measure for types of evidence supporting a PPI, benchmarked against common presence in Kyoto Encyclopedia of Genes and Genomes (KEGG) pathways (Szklarczyk et al. 2023). The median number of types of evidence for each interaction was 19.82. Network statistics ASPL, NC, and BC were computed for each node of the connected network within Cytoscape as it was previously shown that they can partition a network by topology (Wollenberg Valero 2020). Subsequently, the supplementary table from Christmas et al. (2023) containing the constraint metric mean Phylop was merged to the node table to enable comparisons of human-derived network statistics with mammalian constraint measures per gene. Following simulation of additional training data with Synthetic Minority Oversampling TEchnique (SMOTE; Siriseriwan 2022), support vector machine-based classification (Meyer et al. 2023) was performed on nodes with highest values for these categories preclassified (10% for ASPL and NC and 1% for BC due to relatively lower amount of hub nodes in networks; Lawyer 2015). One thousand replicates of 9-fold cross-validation were performed on training sets and stratified training sets, to exclude the possibility of training set imbalance affecting classification. The full training set was then used to classify all remaining nodes into 3 categories (H, hub nodes; I, intermediate nodes; and P, peripheral nodes as described in Wollenberg Valero (2020)  supplementary fig. S1, Supplementary Material online). Classifications with low decision support values were subsequently declassified, denoted as “uncertain,” and excluded from further analysis (supplementary fig. S2, Supplementary Material online). The response variable mean Phylop was normalized, and a linear model was run to analyze the effect of ASPL, BC, and NC on mean Phylop with Cohen's *F* for effect size estimation. The same analysis was repeated for node categories followed by pairwise post hoc tests and effect size estimation. Secondly, genes with significantly accelerated or decelerated evolutionary rates (rho, ρ), in conjunction with the emergence of the hibernation phenotype, were extracted from the data set of Christmas et al. (Christmas et al. 2023). Ten faster evolving and 8 slower evolving were matched in this network (supplementary table S3, Supplementary Material online). For these genes, node position was calculated to explore the topology of hibernation-relevant genes and their mean Phylop scores. A linear model was fitted to test for the effect of node category and direction of association with evolutionary rate on mean Phylop, followed by post hoc tests and effect size estimation. Graphs were generated in Cytoscape, as well as R. More details on supplementary methods and supplementary results are available in supplementary file S1, Supplementary Material online; the R code can be accessed in supplementary file S2, Supplementary Material online; and the data set is available in supplementary file S3, Supplementary Material online.

## Supplementary Material

msae033_Supplementary_Data

## Data Availability

Data are available publicly and provided in the supplementary materials (supplementary file S1, Supplementary Material online, methods and results; supplementary file S2, Supplementary Material online, R code; supplementary file S3, Supplementary Material online, analysis data set).
