# Supplementary material for: Brief Communication: The Predictable Network Topology of Evolutionary Genomic Constraint
Source: Mol Biol Evol. 2024 Feb 16;41(3):msae033. doi: 10.1093/molbev/msae033 (PMC10906983; doi:10.1093/molbev/msae033)
Supplement: msae033_Supplementary_Data [file msae033_supplementary_data.zip › MBE-23-0612_R1_Supplementary_File1.pdf]

Supplementary File 1

Brief Communication: The predictable network topology of evolutionary genomic constraint

Authors: Katharina C. Wollenberg Valero[1*]

Contains: Supplementary Methods and Results, Supplementary Figures S1-S2 and Supplementary Tables S1 to S10

Supplementary Methods

The response variable "mean Phylop" was tested for outliers using Rosner's test. No outliers were identified. Subsequently, meanPhylop was transformed to normal distribution using orderNorm transformation within the *bestNormalize* package and visualized within the Cytoscape network (Figure 1A). From the high-confidence human interactome, Average Shortest Path Length (ASPL), Betweenness Centrality (BC), and Neighborhood Connectivity (NC) were extracted for each gene in Cytoscape and visualized as a 3D-scatterplot (not shown here but see Supplementary File 2). In order to better visualize the trend of mean Phylop across the network, the data points representing genes (nodes) in the network were reduced in dimensionality. This was done through slicing the coordinate system of ASPL, NC and BC into 300 equidistant cubes and representing each cube by its arithmetic mean of mean Phylop values across all data points located within each cube. Figure 1B shows the average mean Phylop values for each cube.

To test the hypothesis (i) that constraint is predicted by the structure of the network, first the three predictors ASPL, NC and BC were scaled, since their different scale could influence some effect size calculations. Assumptions for fitting a linear model were tested. Residuals did not differ from Normality in a Shapiro-Wilk test of a random subset of 200 residuals (using the maximum possible number of 5000 residuals for this test might have led to p-value inflation (W=0.994, p=0.564). Homogeneity of variances was confirmed by visual inspection of Q-Q plots. Due to the large number of sample points in this analysis, the p-value may suffer from p-value inflation, so effect sizes were calculated for all models (Berner and Amrhein 2022).

To test the hypothesis (ii) that constraint differs between network node categories, being highest in H nodes, intermediate in I-nodes and lowest in P nodes, genes were binned into these three categories using Support Vector Machine classification (*svm* function implemented in the *e1071* R package V1.7-14, (Meyer et al. 2023)). Hub nodes are characterized by highest values in BC, intermediate nodes have highest values in NC and peripheral nodes have the highest values of ASPL (Wollenberg Valero 2020). However, the fact that the distributions of each of these predictors are also skewed towards smaller values, means that there is a scarcity of data points with highest values that can be used to train a model to classify points in the rest of the dataset (Supplementary Figure 1). Furthermore, H nodes are considered rare in networks (Lawyer 2015), so there are both comparatively even less nodes for the training set of this class, and as a result also the imbalance in training data compared to I and P nodes is high albeit for biological reasons. Methods to increase training data or to equally represent categories from the actual dataset such as removal of outliers may therefore lead to wrong classification results in the remaining nodes of the network. Therefore, synthetic data generation or SMOTE (Synthetic Minority Oversampling TEchnique, R package *smotefamily* V.1.3.1) was implemented as a workaround to this problem. In this Machine Learning algorithm, synthetic data points are generated within the distribution of existing datasets to fill low-density regions (Siriseriwan 2022). 10% of each highest NC and ASPL values (n=1694 I, n=1696 P),

and 1% of logBC values (n=170 H) were determined as an initial training set. Then, SMOTE was applied which increased the number of H nodes in the training set to n=680 nodes, I to 6775 nodes, and P to 3392 nodes. 100 replicates of 9-fold cross-validation were then performed for this training set and a stratified version of it with all classes having n=680 observations. Mean balanced accuracies were compared using Kolmogorov-Smirnov tests which were not significant. Therefore, the full training dataset including the synthetic training data was then used to discriminate between H, I and P nodes using support vector machine classification, with a 2-degree polynomial kernel, an error coefficient of coef0=2, and gamma value of 20. The resulting classification is shown in Supplementary Figure 1 with final node category counts (including the initial training set values) of n=348 H, n=12,057 I, and n=4552 P nodes.

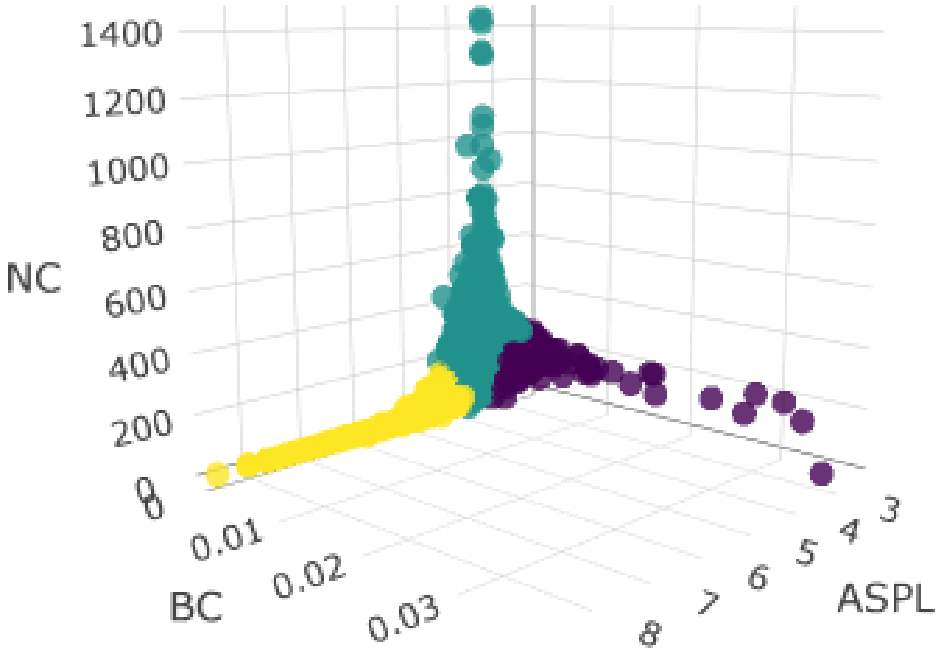

Supplementary Figure 1. Distribution of BC, NC, and ASPL showing nodes with extreme values characterizing H, I and P nodes. Colors represent support vector machine classification results supported by SMOTE simulated training data (H-purple, H - teal, P - yellow).

The accuracy of prediction of the model was evaluated through the decision values to classify each node into one vs. the other two categories. While *svm* decision values for each category can be converted into p-values using multiple ordinal logistic regression, this was not deemed suitable here to assess goodness of prediction due to all node classifications resulting as being significant for each category (not shown). Generally speaking, decision values of ≥ 1 and ≤ -1 are considered distant enough from the hyperplane separating the categories (which in turn is defined by the points themselves) to provide strong support for a node belonging to a category, but there is no hard universal boundary. Here, values of ≥ 0.7 and ≤ -0.7 were used as a cut-off to assess support for categorization, in combination with a similar majority-rule criterion as that used by the *svm*. That is, if less than two out of three decision values were ≥ 0.7 and ≤ -0.7 respectively, the classification was deemed unsupported and the node declassified as “uncertain”. This resulted in a final count of n=306 H, n=11052 I, n=4552 P, and n=1047 uncertain nodes. The distribution of H, I, P and uncertain nodes relative to decision value hyperplanes (through the 0,0,0 coordinates) can be seen in Supplementary

Figure 2. Uncertain nodes were excluded from subsequent statistical analyses. Final classified nodes are visualized within the human interactome in Figure 1C.

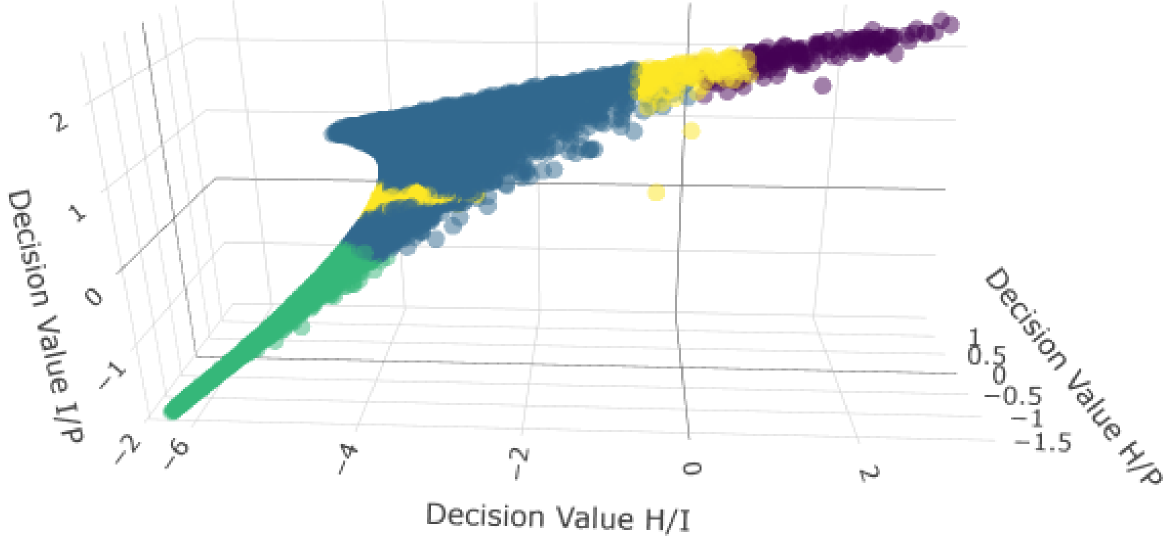

Supplementary Figure 2. Decision values support for nodes classified as H, I, P and uncertain. H - purple, I - teal, P - green, uncertain - yellow.

The next hypothesis (iii) tested was whether hibernation-relevant genes are located in intermediate nodes in the network and differ in constraint values. For this purpose, nodes other than the 18 nodes involved in hibernation with available mean Phylop values and defined node class (n=1 node classified as uncertain) were removed from the dataset. In summary, 1 node was classified as H, 12 as I, and 4 as P. Thus, as expected under hypothesis (iii), the majority of genes whose evolutionary rate changes are significantly associated with the emergence of hibernation phenotype in mammals, were located in I-nodes (Figure 2A). A linear model was then fitted to determine the effect of node class and direction of evolutionary rate association on mean Phylop followed by *post hoc* tests and effect size estimation as described above.

Lastly, hypothesis (iv) was tested that positions of hibernation-relevant genes in the network are not just an outcome of chance. Since node position is here determined by ASPL, BC and NC, the distributions of 1000 iterations of 18 randomly picked genes' ASPL, NC, BC, and mean Phylop, were compared to the respective values of the 18 genes involved in hibernation. Differences in distributions were assessed with Kolmogorov-Smirnov tests for distribution differences between all genes, and additionally for the faster and slower evolving genes separately (but noting that these are each less than n = 18).

Supplementary Results

ANOVA was used to test the hypothesis (i) that constraint estimated by mean Phylop is predicted by the structure of the network. The overall model was significant ($F = 449.6$, $p = < 2.2e-16$, $df=15780$, R^2_{adj}: 0.079), as well as each of the predictors ASPL, NC, and BC (Supplementary Table S1). Due to this model's continuous predictors, Cohen's f was calculated as 0.086 to determine the overall effect

size of the model (or, 8.55% of the variance in mean Phylop being explained by the model). The model intercept was not significant (Supplementary Table S1).

Supplementary Table S1. Coefficients for single predictors for meanPhylop.The overall model was significant at F = 449.6, p= < 2.2e-16, df=15780, R2adj: 0.079.

Predictor	Estimate	Std.Error	t	Pr(>|t|)
(Intercept)	-0.005948	0.007643	-0.778	0.4364
AverageShortestPathLength	-0.326063	0.009263	-35.202	<2e-16
NeighborhoodConnectivity	-0.167005	0.008847	-18.877	<2e-16
BetweennessCentrality	0.018402	0.007599	2.422	0.0155

The hypothesis (ii) whether network node categories determine values of network constraint, was then assessed with an ANOVA, with the overall model significant at F =218.6, p <2e-16, df=14825. Taking into account the categorical nature of predictors in this model, whole-model effect sizes were determined through partial η^2 and partial ω^2 (R package *effectsize*, Ben-Shachar et al. 2020), including the evidence interpretations of η^2 by Cohen (Cohen 1992) and the interpretation of Cohen's d for pairwise *post hoc* tests by Sawilowsky (Sawilowsky 2009). In addition, evidence for p-values is given as: p ≤ 0.1 "Little or none", p ≤ 0.05 "Weak", p ≤ 0.01 "Moderate", p ≤ 0.001 "Strong", p ≤ 0.0001 "Very strong", In this model, there was no significant residual effect, node class overall had a small effect on variance in mean Phylop, with evidence for the effect of node class on mean Phylop being very strong (Supplementary Table S2).

Supplementary Table S2. ANOVA and effect size for analysis of variance for influence of node class on constraint (orderNorm meanPhylop).

term	sumsq	mean sq	df	F	p	partial η^2	partial ω^2	η^2 effect size interpretation	p-value evidence interpretation
Node class (H,I,P)	427	213	2	219	0	0.029	0.029	small	Very strong
Residuals	14499	0.978	14825						

Differences in orderNorm transformed mean Phylop were determined using estimated marginal means *post-hoc* tests (R package *emmeans*, Lenth et al. 2019), where all node classes were found to significantly differ from each other, with meanPhylop being highest in H, lower in I, and lowest in P nodes as shown for yeast in Wollenberg Valero, 2020 (Wollenberg Valero 2020), Supplementary

Table S3, Figure 1D). Pairwise differences in mean Phylop between node categories were significant with large to huge effect on mean Phylop (Supplementary Table S3).

Supplementary Table S3. Test of differences between node classes in orderNorm mean phyloP using estimated marginal means and estimates of effect size.

contrast	estimate	SE	df	t.ratio	emmeans p	d	η^2	η^2 effect size interpretation	Cohen's d evidence interpretation
H - I	0.5052	0.0582	14825	8.6813	0	1.7108	0.4225	large	very large
H - P	0.8364	0.0594	14825	14.0751	0	2.7737	0.6579	large	huge
I - P	0.3312	0.0182	14825	18.1773	0	3.5821	0.7624	large	huge

Supplementary Table S4. Genes with significant evolutionary rate acceleration / deceleration (ρ) associated with hibernating life history strategy from Christmas et al. (Christmas et al. 2023). Significant ρ values already imply a degree of constraint since the rate change has to occur in several lineages simultaneously.

Direction of hibernation ρ	Gene symbol	ρ	mean Phylop	node class
2 faster	HSPD1	0.284938329	4.681327293	Hub
2 faster	CRK	0.408376322	6.212137001	Intermediate
2 faster	HNRNPUL1	0.212523592	4.98841049	Intermediate
2 faster	SCN2A	0.230656402	4.823024502	Intermediate
2 faster	CHSY3	0.220281753	4.234399474	Intermediate
2 faster	ADAMTS9	0.288636113	3.858180248	Intermediate
2 faster	DENND6A	0.201455581	4.917183534	Uncertain
2 faster	SLC12A5	0.239446422	5.374007236	Intermediate
2 faster	KLHL14	0.317113808	5.579078687	Peripheral
2 faster	CCDC105	0.193813592	2.504036649	Peripheral
1 slower	CENPJ	-0.266986284	2.283312178	Intermediate
1 slower	RIOK2	-0.233911687	3.497984108	Intermediate
1 slower	ALDH6A1	-0.270680198	4.845277778	Intermediate
1 slower	RAB22A	-0.346481353	4.529034258	Intermediate
1 slower	EXD1	-0.222540581	2.567539432	Peripheral
1 slower	KRT78	-0.25622918	2.493554096	Peripheral
1 slower	ARMC9	-0.210691253	3.241726596	Intermediate
1 slower	ITGBL1	-0.241240487	4.156428385	Intermediate

To test hypothesis (iii) whether the direction of association (evolutionary rate accelerating or slowing in response to the emergence of a hibernation phenotype), or the node class were associated with constraint values in these genes, a linear model was fitted followed by *post hoc* tests and effect size estimation as described above. The node class did not significantly influence constraint (possibly due to the majority of genes being located in I-nodes), but faster evolving genes had higher constraint value than slower evolving genes, with large effect size and moderate statistical support

(Supplementary Table S5). The pairwise differences in mean Phylop between fast and slow genes were small but with significant statistical support.

Supplementary Table S5. ANOVA and effect size for analysis of variance for influence of node category and direction of rate association on constraint (orderNorm mean Phylop) in hibernation-associated genes.

term	sumsq	meansq	df	F	P	partial. η^2	partial ω^2	η^2 effect size interpretation	p-value evidence interpretation
Direction	5.163	5.163	1	6.229	0.027	0.324	0.235	large	Moderate
Node class	1.545	0.773	2	0.932	0.419	0.125	-0.008	small	Little or none
Residuals	10.776	0.829	13	NA	NA	NA	NA	NA	NA

Supplementary Table S6. Test of differences in directions of evolutionary rate association with hibernation, and orderNorm mean Phylop using estimated marginal means and estimates of effect size.

contrast	estimate	SE	df	t.ratio	emmeans p	d	η^2	η^2 effect size interpretation	Cohen's d evidence interpretation
slower - faster	-1.118	0.427	14	-2.619	0.02	-0.516	0.062	small	medium

Lastly, hypothesis (iv), that the predominance of intermediate positions of hibernation-relevant genes in the network is not just an outcome of chance was tested. All three predictors (ASPL, NC and BC as well as mean Phylop) significantly differed between genes associated with hibernation and those obtained by 1000 replicates of 18 randomly picked genes, showing that the position of the nodes in the network is not just an outcome of chance due to predominance of parameters defining I-nodes and their large number within the interactome (Supplementary Tables S7-S10). This effect could be mostly due to faster-evolving genes, but due to differences in sample size between faster and slower evolving genes, this cannot be conclusively stated here.

Supplementary Table S7. Results of Kolmogorov-Smirnov tests for differences in ASPL between 1000 randomly drawn sets of 18 genes, and 18 genes associated with hibernation in mammals as well as the subsets of slower and faster evolving genes.

Test	D statistic	p value
hibernation genes vs. simulated means	0.332	0.040
slower vs. simulated means	0.374	0.168
faster vs. simulated means	0.300	0.335

Supplementary Table S8. Results of Kolmogorov-Smirnov tests for differences in BC between 1000 randomly drawn sets of 18 genes, and 18 genes associated with hibernation in mammals as well as the subsets of slower and faster evolving genes.

Test	D statistic	p value
hibernation genes vs. simulated means	0.457	0.001
slower vs. simulated means	0.500	0.024
faster vs. simulated means	0.501	0.014

Supplementary Table S9. Results of Kolmogorov-Smirnov tests for differences in NC between 1000 randomly drawn sets of 18 genes, and 18 genes associated with hibernation in mammals as well as the subsets of slower and faster evolving genes.

Test	D statistic	p value
hibernation genes vs. simulated means	0.499	0.0003
slower vs. simulated means	0.382	0.151
faster vs. simulated means	0.654	0.0004

Supplementary Table S10. Results of Kolmogorov-Smirnov tests for differences in mean Phylop (not normalized) between 1000 randomly drawn sets of 18 genes, and 18 genes associated with hibernation in mammals as well as the subsets of slower and faster evolving genes.

Test	D statistic	p value
hibernation genes vs. simulated means	0.581	0.000007
slower vs. simulated means	0.364	0.203
faster vs. simulated means	0.785	0.0000009

Supplementary References

Ben-Shachar M, Lüdecke D, Makowski D. 2020. Effectsize: Estimation of effect size indices and standardized parameters. *J. Open Source Softw.* 5:2815.

Berner D, Amrhein V. 2022. Why and how we should join the shift from significance testing to estimation. *J. Evol. Biol.* 35:777–787.

Christmas MJ, Kaplow IM, Genereux DP, Dong MX, Hughes GM, Li X, Sullivan PF, Hindle AG, Andrews G, Armstrong JC, et al. 2023. Evolutionary constraint and innovation across hundreds of placental mammals. *Science* 380:eabn3943.

Cohen J. 1992. A power primer. *Psychol. Bull.* 112:155–159.

Lawyer G. 2015. Understanding the influence of all nodes in a network. *Sci. Rep.* 5:8665.

Lenth R, Singmann H, Love J, Buerkner P, Herve M. 2019. Package "emmeans." *R package version*.

Meyer D, Dimitriadou E, Hornik K, Weingessel A, Leisch F, Chang C-C, Lin C-C. 2023. Package "e1071." Available from: https://cran.r-project.org/web/packages/e1071/e1071.pdf

Sawilowsky SS. 2009. New Effect Size Rules of Thumb. *J. Mod. Appl. Stat. Methods* 8:26.

Siriseriwan W. 2022. Package "smotefamily." Available from: https://cran.r-project.org/web/packages/smotefamily/smotefamily.pdf

Wollenberg Valero KC. 2020. Aligning functional network constraint to evolutionary outcomes. *BMC Evol. Biol.* 20:58.
