# Supplementary material for: Brief Communication: The Predictable Network Topology of Evolutionary Genomic Constraint
Source: Mol Biol Evol. 2024 Feb 16;41(3):msae033. doi: 10.1093/molbev/msae033 (PMC10906983; doi:10.1093/molbev/msae033)
Supplement: msae033_Supplementary_Data [file msae033_supplementary_data.zip › MBE-23-0612_R1_Supplementary_File2.html]

Analysis of network constraint in mammals


# Analysis of network constraint in mammals

#### Kat Valero

#### 2023-12-22

- Script
  for analyzing genomic network constraint at the protein coding gene
  level in placental mammals (on the example of the human
  network)
- Data
  preprocessing
- Visualization of
  constraint in the network
- Test
  of Hypothesis 1: constraint is predicted by the structure of the
  network
  - Result of ANOVA and effect
    sizes
- Test
  of Hypothesis 2: constraint differs between network node categories,
  being highest in H nodes, intermediate in I-nodes and lowest in P
  nodes.
  - Initial classification
  - Simulation of training
    data using SMOTE
  - Node
    classification with Support Vector Machine (SVM)
  - Declassification
    of Uncertain identity nodes
  - Result of ANOVA and effect
    sizes
- Test
  of Hypothesis 3. Hibernation-relevant genes are located in intermediate
  nodes and differ in constraint values
  - Effect
    of Node category and direction of rate change rho on
    Hibernation-relevant genes
- Test
  of Hypothesis 4. The intermediate position of hibernation-relevant genes
  in the network is not just an outcome of chance due to predominance of
  this node category within the interactome.
  - Tests for Average
    Shortest Path Length
  - Tests for Betweenness
    Centrality
  - Tests for Neighborhood
    Connectivity
  - Test for
    meanPhylop

### Script for analyzing genomic network constraint at the protein coding gene level in placental mammals (on the example of the human network)

The data on constraint was obtained from: Christmas, M. J., Kaplow,
I. M., Genereux, D. P., Dong, M. X., Hughes, G. M., Li, X., Sullivan, P.
F., Hindle, A. G., Andrews, G., Armstrong, J. C., Bianchi, M., Breit, A.
M., Diekhans, M., Fanter, C., Foley, N. M., Goodman, D. B., Goodman, L.,
Keough, K. C., Kirilenko, B., … Karlsson, E. K. (2023). Evolutionary
constraint and innovation across hundreds of placental mammals. Science,
380(6643), eabn3943.

# Data preprocessing

set the working directory, open the data and load packages.

```
setwd("C:/Users/kcwol/Downloads/mammalperspective_viperdata")
df <- read.csv("C:/Users/kcwol/Downloads/mammalperspective_viperdata/mammals_stats6_nodfa.csv")

library(plotly)
library(webshot)
library(ggplot2)
library(dplyr)
library(orca)
library(bestNormalize)
library(effectsize)
library(emmeans)
library(dunn.test)
library(broom)
library(rstatix)
library(scico)
library(sjstats)
library(kableExtra)
library(EnvStats)
library(Hmisc)
library(smotefamily)
library(MASS)
library(e1071)
library(caTools)
library(nnet)
library(caret)
library(viridis)
library(scales)
```

Check for outliers in the response variable mean Phylop

Figure. Boxplot of mean Phylop

Determine whether there are any outliers in the response variable

```
test <- rosnerTest(df$meanPhylop,
  k = 10)
test
```

```
## 
## Results of Outlier Test
## -------------------------
## 
## Test Method:                     Rosner's Test for Outliers
## 
## Hypothesized Distribution:       Normal
## 
## Data:                            df$meanPhylop
## 
## Number NA/NaN/Inf's Removed:     1173
## 
## Sample Size:                     15784
## 
## Test Statistics:                 R.1  = 4.010473
##                                  R.2  = 3.884635
##                                  R.3  = 3.836776
##                                  R.4  = 3.699596
##                                  R.5  = 3.683630
##                                  R.6  = 3.651400
##                                  R.7  = 3.582222
##                                  R.8  = 3.545119
##                                  R.9  = 3.539957
##                                  R.10 = 3.526327
## 
## Test Statistic Parameter:        k = 10
## 
## Alternative Hypothesis:          Up to 10 observations are not
##                                  from the same Distribution.
## 
## Type I Error:                    5%
## 
## Number of Outliers Detected:     0
## 
##    i   Mean.i     SD.i     Value Obs.Num    R.i+1 lambda.i+1 Outlier
## 1  0 3.500269 1.438392 -2.268365    2678 4.010473   4.658103   FALSE
## 2  1 3.500634 1.437705 -2.084323     418 3.884635   4.658090   FALSE
## 3  2 3.500988 1.437063 -2.012699    9721 3.836776   4.658076   FALSE
## 4  3 3.501338 1.436438 -1.812902    2571 3.699596   4.658063   FALSE
## 5  4 3.501675 1.435860 -1.787503    5622 3.683630   4.658050   FALSE
## 6  5 3.502010 1.435288 -1.738800    1589 3.651400   4.658037   FALSE
## 7  6 3.502342 1.434727 -1.637168   14664 3.582222   4.658024   FALSE
## 8  7 3.502668 1.434189 -1.581702    1115 3.545119   4.658011   FALSE
## 9  8 3.502990 1.433663 -1.572114     714 3.539957   4.657998   FALSE
## 10 9 3.503312 1.433138 -1.550403    1953 3.526327   4.657985   FALSE
```

There are no outliers. Make a histogram for mean PhyloP:

```
plot <- ggplot(df, aes(x = meanPhylop)) +
  geom_histogram(binwidth = 0.1, fill = "#002051", color = "black") +
  labs(title = "Distribution of median of the Mean PhyloP Values") + theme_minimal()
# Save the histogram as a PDF
ggsave("Hist_meanphylop._untransformed.pdf", width = 10, height = 10)
```

```
## Warning: Removed 1173 rows containing non-finite values (`stat_bin()`).
```

Determine best method for normal transformation of response
variable

```
df$meanPhylop <- as.numeric(df$meanPhylop)
(bn_meanPhylop <- bestNormalize(df$meanPhylop))
```

```
## Warning: `progress_estimated()` was deprecated in dplyr 1.0.0.
## ℹ The deprecated feature was likely used in the bestNormalize package.
##   Please report the issue to the authors.
## This warning is displayed once every 8 hours.
## Call `lifecycle::last_lifecycle_warnings()` to see where this warning was
## generated.
```

```
## Best Normalizing transformation with 15784 Observations
##  Estimated Normality Statistics (Pearson P / df, lower => more normal):
##  - arcsinh(x): 28.3016
##  - Center+scale: 7.4771
##  - Double Reversed Log_b(x+a): 1.6494
##  - Exp(x): 45.2308
##  - Log_b(x+a): 18.3097
##  - orderNorm (ORQ): 1.1137
##  - sqrt(x + a): 11.7974
##  - Yeo-Johnson: 3.2102
## Estimation method: Out-of-sample via CV with 10 folds and 5 repeats
##  
## Based off these, bestNormalize chose:
## orderNorm Transformation with 15784 nonmissing obs and ties
##  - 15779 unique values 
##  - Original quantiles:
##     0%    25%    50%    75%   100% 
## -2.268  2.669  3.761  4.580  6.762
```

Transform the response variable with orderNorm transformation. This
snippet may be repeated below as datasets are split, merged and
otherwise transformed.

```
BN_meanPhylop <- orderNorm(df$meanPhylop)
orderNorm_meanPhylop <- predict(BN_meanPhylop)
df <- cbind(df, orderNorm_meanPhylop)
```

Make a histogram for order Norm transformed dependent variable “mean
PhyloP”

```
ggplot(df, aes(x = orderNorm_meanPhylop)) +
  geom_histogram(binwidth = 0.1, fill = "#fdea45", color = "black") +
  labs(title = "Distribution of median orderNorm_Mean PhyloP Values") + theme_minimal()
```

```
ggsave("Hist_meanphylop_oNtransformed.pdf", width = 10, height = 10)
```

# Visualization of constraint in the network

We use Average Shortest Path Length (ASPL), BetweeennessCentrality
(BC), and Neighborhood Connectivity (NC). These values have been
extracted from the human interactome using the network analysis function
in Cytoscape

Create 3D scatterplot for ASPL, BC, and NC with R (plotly library) to
see variation in order Norm transformed mean Phylop

```
df$orderNorm_meanPhylop <- as.numeric(df$orderNorm_meanPhylop)
NWstats_meanphylop_3Dscatter <- plot_ly(df, type = "scatter3d", mode = "markers",
               x = ~AverageShortestPathLength, y = ~BetweennessCentrality, z = ~NeighborhoodConnectivity,
               marker = list(size = 8, opacity = 0.5, color = ~orderNorm_meanPhylop, colorscale = 'Cividis',colorbar = list(title = "oN mean PhyloP", tickmode = "auto", ticks = "outside", ticklen = 10), text = ~paste("oN_meanPhylop: ", orderNorm_meanPhylop))) %>%
  layout(scene = list(xaxis = list(type = "log", title = "log_ASPL"),
                      yaxis = list(type = "log", title = "log_BC"),
                      zaxis = list(type = "log", title = "log_NC")))
NWstats_meanphylop_3Dscatter
```

Reduce the dimensionality of the data to better visualize the trend
of constraint values changing across the network (to n=300 points), by
slicing it into equidistant cubes and calculating mean values of
(oN\_mean\_Phylop) per each cube

```
#make dataframe copy
original_data <- df

num_final_points <- 300
num_cubes <- round(num_final_points^(1/3))
x_breaks <- quantile(original_data$AverageShortestPathLength, probs = seq(0, 1, length.out = num_cubes + 1), na.rm = TRUE)
y_breaks <- quantile(original_data$BetweennessCentrality, probs = seq(0, 1, length.out = num_cubes + 1), na.rm = TRUE)
z_breaks <- quantile(original_data$NeighborhoodConnectivity, probs = seq(0, 1, length.out = num_cubes + 1), na.rm = TRUE)

original_data$Cube <- cut(original_data$AverageShortestPathLength, breaks = x_breaks, labels = FALSE) +
                     num_cubes * cut(original_data$BetweennessCentrality, breaks = y_breaks, labels = FALSE) +
                     num_cubes^2 * cut(original_data$NeighborhoodConnectivity, breaks = z_breaks, labels = FALSE)
```

Create a 3D scatter plot of the cube identities for all points

```
cube_figure <- plot_ly(original_data, type = "scatter3d", mode = "markers",
               x = ~AverageShortestPathLength, y = ~BetweennessCentrality, z = ~NeighborhoodConnectivity,
               marker = list(size = 8, opacity = 0.5, color = ~Cube, colorscale = 'Viridis', colorbar = list(title = "oN mean PhyloP", tickmode = "auto", ticks = "outside", ticklen = 10), text = ~paste("Cube_number: ", Cube))) %>%
  layout(scene = list(xaxis = list(type = "log", title = "log ASPL"),
                      yaxis = list(type = "log", title = "log BC"),
                      zaxis = list(type = "log", title = "log NC")))
cube_figure
```

Bin each cube’s datapoints into one average point using means of
coordinates

```
mean_cube_coordinates <- aggregate(cbind(AverageShortestPathLength, BetweennessCentrality, NeighborhoodConnectivity) ~ Cube, data = original_data, mean, na.rm = TRUE)

binned_cube_figure <- plot_ly(mean_cube_coordinates, type = "scatter3d", mode = "markers",
                   x = ~AverageShortestPathLength, y = ~BetweennessCentrality, z = ~NeighborhoodConnectivity,
                   marker = list(size = 8, opacity = 0.5, color = ~Cube, colorscale = 'Viridis', colorbar = list(title = "Cube bins", tickmode = "auto", ticks = "outside", ticklen = 10), text = ~paste("Cube: ", Cube))) %>%
  layout(scene = list(xaxis = list(type = "log", title = "log ASPL"),
                      yaxis = list(type = "log", title = "log BC"),
                      zaxis = list(type = "log", title = "log NC")))

binned_cube_figure
```

calculate the average order Norm transformed mean Phylop for each
cube

```
mean_phylop_coordinates_per_cube_mean <- aggregate(cbind(AverageShortestPathLength, BetweennessCentrality, NeighborhoodConnectivity, meanPhylop, orderNorm_meanPhylop) ~ Cube, data = original_data, mean, na.rm = TRUE)

#save this new datasheet to csv
write.csv(original_data,"mammals_stats_cubed2.csv", row.names=FALSE)
```

also flip the axis so that smallest ASPL meets largest BC to better
represent the source network as: outside (large ASPL, small BC)
vs. inside (small ASPL, large BC). Filter out one outlier in BC and plot
log values

```
#subset dataframe
filtered_data <- mean_phylop_coordinates_per_cube_mean %>%
  filter(BetweennessCentrality < 0.002)

color_scale_min <- quantile(filtered_data$orderNorm_meanPhylop, probs = 0.1)
color_scale_max <- quantile(filtered_data$orderNorm_meanPhylop, probs = 0.8)
Binned_NWstats_meanphylop_3Dscatter <- plot_ly(filtered_data, type = "scatter3d", mode = "markers",
                                  x = ~AverageShortestPathLength, y = ~BetweennessCentrality, z = ~NeighborhoodConnectivity,
                                  marker = list(size = 8, opacity = 0.5, 
                                                color = ~orderNorm_meanPhylop, 
                                                colorscale='Cividis', 
                                                cmin = color_scale_min, 
                                                cmax = color_scale_max,
                                                colorbar = list(title = "oN Mean PhyloP", tickmode = "auto", ticks = "outside", ticklen = 10))) %>%
  layout(scene = list(xaxis = list(type = "linear", title = "ASPL", autorange="reversed"),
                      yaxis = list(type = "log", title = "BC", autorange="reversed"),
                      zaxis = list(type = "linear", title = "NC")))

Binned_NWstats_meanphylop_3Dscatter
```

Admire how the points get lighter/more yellow from the outside
towards the center…

# Test of Hypothesis 1: constraint is predicted by the structure of the network

Make Histograms for NC, BC, and ASPL

```
ggplot(original_data, aes(x = AverageShortestPathLength)) +
  geom_histogram(binwidth = 0.1, fill = "#002051", color = "black") +
  labs(title = "Distribution of ASPL")
```

```
ggplot(original_data, aes(x = NeighborhoodConnectivity)) +
  geom_histogram(binwidth = 1, fill = "azure4", color = "black") +
  labs(title = "Distribution of NC")
```

```
ggplot(original_data, aes(x = BetweennessCentrality)) +
  geom_histogram(binwidth = 0.001, fill = "#fdea45", color = "black") +
  labs(title = "Distribution of BC")
```

These predictors are on totally different scales which may influence
some effect size calculations later. So they will first be scaled.

```
#make new dataframe 
original_data2 <- original_data

original_data2$AverageShortestPathLength <- scale(original_data2$AverageShortestPathLength)
original_data2$NeighborhoodConnectivity <- scale(original_data2$NeighborhoodConnectivity)
original_data2$BetweennessCentrality <- scale(original_data2$BetweennessCentrality)

#save as new datasheet
write.csv(original_data2, "mammals_stats_scaled.csv", row.names=FALSE)
```

Test model assumptions and fit a linear model lm for the effect of
ASPL, BC and NC on orderNorm transformed mean Phylop

```
model <- lm(orderNorm_meanPhylop ~ AverageShortestPathLength + NeighborhoodConnectivity + BetweennessCentrality, data = original_data2)
model.metrics <- augment(model)
model.metrics <- model.metrics[, !(names(model.metrics) %in% c(".hat", ".sigma", ".fitted"))]
head(model.metrics, 3)
```

```
## # A tibble: 3 × 8
##   .rownames orderNorm_meanPhylop AverageShortestPathLen…¹ NeighborhoodConnecti…²
##   <chr>                    <dbl>                    <dbl>                  <dbl>
## 1 1                        0.198                    -1.50                 0.0970
## 2 2                       -1.36                     -2.02                 0.517 
## 3 3                       -1.03                     -1.78                 0.337 
## # ℹ abbreviated names: ¹​AverageShortestPathLength[,1],
## #   ²​NeighborhoodConnectivity[,1]
## # ℹ 4 more variables: BetweennessCentrality <dbl[,1]>, .resid <dbl>,
## #   .cooksd <dbl>, .std.resid <dbl>
```

Run a Shapiro test for deviations of residuals from Normality. The
max rows for this are exceeded with the full dataset and to prevent
p-value inflation, here only a subset of random rows is tested.

```
sampled_resid <- sample(model.metrics$.resid, 200)
shapiro_test(sampled_resid)
```

```
## # A tibble: 1 × 3
##   variable      statistic p.value
##   <chr>             <dbl>   <dbl>
## 1 sampled_resid     0.994   0.564
```

The residuals don’t deviate from Normality Make a Q-Q plot for
visually checking homogeneity of variances

```
qqnorm(sampled_resid)
qqline(sampled_resid)
```

## Result of ANOVA and effect sizes

Display the summary of the model

```
summary(model)
```

```
## 
## Call:
## lm(formula = orderNorm_meanPhylop ~ AverageShortestPathLength + 
##     NeighborhoodConnectivity + BetweennessCentrality, data = original_data2)
## 
## Residuals:
##     Min      1Q  Median      3Q     Max 
## -4.0997 -0.6415 -0.0084  0.6227  4.5214 
## 
## Coefficients:
##                            Estimate Std. Error t value Pr(>|t|)    
## (Intercept)               -0.005948   0.007643  -0.778   0.4364    
## AverageShortestPathLength -0.326063   0.009263 -35.202   <2e-16 ***
## NeighborhoodConnectivity  -0.167005   0.008847 -18.877   <2e-16 ***
## BetweennessCentrality      0.018402   0.007599   2.422   0.0155 *  
## ---
## Signif. codes:  0 '***' 0.001 '**' 0.01 '*' 0.05 '.' 0.1 ' ' 1
## 
## Residual standard error: 0.9599 on 15780 degrees of freedom
##   (1173 observations deleted due to missingness)
## Multiple R-squared:  0.07874,    Adjusted R-squared:  0.07857 
## F-statistic: 449.6 on 3 and 15780 DF,  p-value: < 2.2e-16
```

To estimate overall effect size, calculate Cohen’s f squared for the
entire model which is ratio of the explained variance to the residual
variance

```
R_squared <- summary(model)$r.squared
f_squared <- R_squared / (1 - R_squared)
f_squared
```

```
## [1] 0.08547111
```

Calculate effect sizes for each predictor as Partial Eta-squared
(η²), representing the proportion of variance in the dependent variable
that is associated with a particular predictor, while controlling for
the effects of other predictors in the model.

```
eta_squared <- eta_squared(model)
eta_squared
```

```
## AverageShortestPathLength  NeighborhoodConnectivity     BetweennessCentrality 
##              0.0571201025              0.0212785302              0.0003423979
```

# Test of Hypothesis 2: constraint differs between network node categories, being highest in H nodes, intermediate in I-nodes and lowest in P nodes.

To visualise node position within the network better, nodes are
categorized based on ASPL, BC and NC. Categories are Hub, Peripheral and
Intermediate H, P and I.

Problem: The distributions of ASPL, BC and NC show skew, but most
extreme values are not represented by large numbers of datapoints. For
example, Hub nodes are characterised by large Betweeness Centrality, but
the number of true hub nodes in the dataset is expected to be
proportionally rather small. Therefore, methods to increase training
data or to equally represent categories from the actual dataset such as
removal of outliers or stratification, may lead to wrong classification
results. We can implement a workaround called “synthetic data
generation” or SMOTE (Synthetic Minoriry oversampling technique) which
is a Machine Learning algorithm. Here, lines are drawn between two
datapoints in the original dataset and new synthetic datapoints are
generated along that line. This way, the areas of the distribution with
low dots are “filled up”. The k parameter ensures this is only done
between close neighbors to avoid deviating too much from the original
distribution when doing so.

## Initial classification

First we perform an initial classification, where nodes with extreme
values of ASPL, BC and NC get binned into H, I and P categories. Note
that less BC values are taken due to its extremely skewed
distribution.

```
#open new dataset here just as a starting point to be able to repeat this a few times without having to delete out any columns. Otherwise commenting this out and continuing with the main analysis dataset is fine. 
dataset <- read.csv('mammals_stats7_nodfa.csv')

dataset$id <- seq_len(nrow(dataset))
dataset$DFA_Nodecat <- NA
#using log BC here because of its underlying distribution causing issues with classification based on percentiles
dataset$log_BetweennessCentrality <- log(dataset$BetweennessCentrality + 1)  # +1 to avoid log(0)
threshold_H <- quantile(dataset$log_BetweennessCentrality, probs = 0.99, na.rm = TRUE)
threshold_I <- quantile(dataset$NeighborhoodConnectivity, probs = 0.9, na.rm = TRUE) 
threshold_P <- quantile(dataset$AverageShortestPathLength, probs = 0.9, na.rm = TRUE) 

dataset$DFA_Nodecat <- ifelse(dataset$log_BetweennessCentrality > threshold_H, 'H',
                       ifelse(dataset$NeighborhoodConnectivity > threshold_I, 'I',
                               ifelse(dataset$AverageShortestPathLength > threshold_P, 'P', NA)))

dataset$DFA_Nodecat <- as.factor(dataset$DFA_Nodecat)
print("table of initial node category counts")
```

```
## [1] "table of initial node category counts"
```

```
table(dataset$DFA_Nodecat)
```

```
## 
##    H    I    P 
##  170 1694 1696
```

```
train_set <- dataset[!is.na(dataset$DFA_Nodecat), ]
test_set <- dataset[is.na(dataset$DFA_Nodecat), ]

print("row count of train_set")
```

```
## [1] "row count of train_set"
```

```
print(nrow(train_set))
```

```
## [1] 3560
```

```
print("row count of test_set")
```

```
## [1] "row count of test_set"
```

```
print(nrow(test_set))
```

```
## [1] 13397
```

```
# Create a copy of this initially classified data for later use
initial_classified_copy <- train_set[!is.na(train_set$DFA_Nodecat), ]
```

## Simulation of training data using SMOTE

SMOTE by default is applied to binary class problems - needs to be
optimized for multi class problem (such as our H,I,P nodes). The
majority class in train\_set$DFA\_Nodecat is first identified to
distinguish it from the minority classes and then a loop iterates over
each class level in DFA\_Nodecat. For each class (except the majority
class), a binary classification dataset is created, where the current
class is treated as one class and all other classes are combined into a
second class (“Other”). This is done to apply SMOTE separately to each
minority class against the rest of the data.Then everything is merged
back together to create a balanced training set.

```
train_set$DFA_Nodecat <- as.factor(train_set$DFA_Nodecat)
majority_class <- names(sort(table(train_set$DFA_Nodecat), decreasing = TRUE))[1]
balanced_train_set <- data.frame()

for (class in levels(train_set$DFA_Nodecat)) {
  if (class != majority_class) {
    binary_data <- train_set[, c('AverageShortestPathLength','BetweennessCentrality', 'log_BetweennessCentrality', 'NeighborhoodConnectivity')]
    binary_target <- as.factor(ifelse(train_set$DFA_Nodecat == class, class, "Other"))
    smote_data <- SMOTE(binary_data[, c('AverageShortestPathLength','BetweennessCentrality', 'NeighborhoodConnectivity')], 
                        binary_target, 
                        K = 5,
                        dup_size = 2) 
    if (is.list(smote_data) && "data" %in% names(smote_data)) {
      combined_data <- smote_data$data
      combined_data$DFA_Nodecat <- as.factor(combined_data$class)
      balanced_train_set <- rbind(balanced_train_set, combined_data[combined_data$DFA_Nodecat == class, ])
    }
  }
}

balanced_train_set$class <- NULL

print("row count of balanced_train_set")
```

```
## [1] "row count of balanced_train_set"
```

```
print(nrow(balanced_train_set))
```

```
## [1] 5592
```

```
common_columns <- intersect(names(train_set), names(balanced_train_set))
initial_classified_copy2 <- train_set[!is.na(train_set$DFA_Nodecat), common_columns]
majority_class_data <- train_set[train_set$DFA_Nodecat == majority_class, common_columns, drop = FALSE]
balanced_train_set <- rbind(balanced_train_set, majority_class_data)

print("row count of balanced_train_set after binding majority data")
```

```
## [1] "row count of balanced_train_set after binding majority data"
```

```
print(nrow(balanced_train_set))
```

```
## [1] 7288
```

```
balanced_train_set <- balanced_train_set[balanced_train_set$DFA_Nodecat != "Other", ]
balanced_train_set$DFA_Nodecat <- droplevels(balanced_train_set$DFA_Nodecat)
balanced_train_set <- rbind(balanced_train_set, initial_classified_copy2)

print("Row count of balanced_train_set after adding initial_classified_copy:")
```

```
## [1] "Row count of balanced_train_set after adding initial_classified_copy:"
```

```
print(nrow(balanced_train_set))
```

```
## [1] 10848
```

```
table(balanced_train_set$DFA_Nodecat)
```

```
## 
##    H    I    P 
##  680 6776 3392
```

```
print("row count of DFA_Nodecat in balanced_train_set after SMOTE and exclude other")
```

```
## [1] "row count of DFA_Nodecat in balanced_train_set after SMOTE and exclude other"
```

```
print(nrow(balanced_train_set))
```

```
## [1] 10848
```

## Node classification with Support Vector Machine (SVM)

Run SVM to assign nodes into categories based on ASPL, NC and BC. A
polynomial Kernel is picked to better discriminate nodes based on the
three predictors than a linear DFA would.

```
test_set <- dataset[is.na(dataset$DFA_Nodecat), ] #just make this again, in case some things got messed up along the way
test_set_for_prediction <- test_set[, c('AverageShortestPathLength','BetweennessCentrality', 'BetweennessCentrality','NeighborhoodConnectivity')]


svm_model <- svm(DFA_Nodecat ~ AverageShortestPathLength + BetweennessCentrality + NeighborhoodConnectivity, data = balanced_train_set, 
               kernel = "polynomial", degree = 2, coef0 = 2, gamma = 20)
svm_predictions <- predict(svm_model, newdata = test_set_for_prediction)
svm_predictions_vector <- as.vector(svm_predictions)
print(head(svm_predictions_vector))
```

```
## [1] "H" "I" "I" "I" "I" "I"
```

```
# Attach predictions back to the test_set and copy everything back together
test_set$Predicted_Class2 <- NA
test_set$Predicted_Class2 <- svm_predictions_vector
print("row count of test set_predicted class2 still missing the original training data")
```

```
## [1] "row count of test set_predicted class2 still missing the original training data"
```

```
print(nrow(test_set))
```

```
## [1] 13397
```

```
initial_classified_copy$Predicted_Class2 <- NULL
initial_classified_copy$Predicted_Class2 <- NA
initial_classified_copy$DFA_Nodecat <- as.factor(initial_classified_copy$DFA_Nodecat)

initial_classified_copy$Predicted_Class2 <- as.factor(initial_classified_copy$Predicted_Class2)
test_set$Predicted_Class2 <- as.factor(test_set$Predicted_Class2)

test_set <- rbind(test_set, initial_classified_copy)
test_set$Predicted_Class2[is.na(test_set$Predicted_Class2)] <- test_set$DFA_Nodecat[is.na(test_set$Predicted_Class2)]
test_set <- unique(test_set)

print("table of node category counts after SVM and adding training data")
```

```
## [1] "table of node category counts after SVM and adding training data"
```

```
print(nrow(test_set))
```

```
## [1] 16957
```

```
table(test_set$Predicted_Class2)
```

```
## 
##     H     I     P 
##   348 12057  4552
```

It is not possible to validate this model since the initial training
set was used to absolutely distinguish the categories, so if we would
back- predict these with the balanced training set-derived model, the
model will artificially look perfect due to it containing a circular
argument. We can instead look at the classification decision values
(further down), and visually inspect how points were classified based on
ASPL, BC and NC.

Make a 3D scatterplot of the classification outcome

```
plot_ly(data = test_set, x = ~AverageShortestPathLength, y = ~BetweennessCentrality, z = ~NeighborhoodConnectivity, 
        type = 'scatter3d', mode = 'markers',
        marker = list(size = 5, color = ~as.numeric(Predicted_Class2), colorscale = 'Viridis', opacity = 0.8),
        text = ~paste('Predicted_Class2:', Predicted_Class2)) %>%
    layout(title = '3D Scatterplot of scaled ASPL, BC,NC',
           scene = list(xaxis = list(title = 'ASPL'),
                        yaxis = list(title = 'BC'),
                        zaxis = list(title = 'NC')))
```

Visually compare constraint values between node categories through a
Means-with-error box plot on both normalized and non-normalized mean
Phylop

```
original_data2 <- test_set
ON_meanPhylop <- orderNorm(original_data2$meanPhylop)
orderNorm_meanPhylop <- predict(ON_meanPhylop)
original_data2 <- cbind(original_data2, orderNorm_meanPhylop)
plot_data <- original_data2

lower_quantile <- quantile(plot_data$orderNorm_meanPhylop, probs = 0.05, na.rm = TRUE)
upper_quantile <- quantile(plot_data$orderNorm_meanPhylop, probs = 0.95, na.rm = TRUE)

assign_combined_color <- function(value, lower_quantile, upper_quantile, lower_color, upper_color, middle_color_scale) {
  if (is.na(value)) {
    return(NA)  # Keep NA if the value is NA
  } else if (value <= lower_quantile) {
    return(lower_color)
  } else if (value >= upper_quantile) {
    return(upper_color)
  } else {
    normalized_value <- (value - lower_quantile) / (upper_quantile - lower_quantile)
    return(middle_color_scale(normalized_value))
  }
}

middle_color_scale <- scales::col_numeric(palette = viridis_pal(option = "cividis")(100), domain = c(0, 1))

plot_data$combined_color <- mapply(assign_combined_color,
                                        plot_data$orderNorm_meanPhylop,
                                        MoreArgs = list(
                                          lower_quantile = lower_quantile,
                                          upper_quantile = upper_quantile,
                                          lower_color = "#002051",
                                          upper_color = "#fdea45",
                                          middle_color_scale = middle_color_scale
                                        ))

Nodecat_on_meanphylop_meplot_svm <- ggplot(plot_data, aes(x = Predicted_Class2, y = orderNorm_meanPhylop)) +
  geom_jitter(aes(colour = combined_color), alpha = 0.3, shape = 16, size=4) +
  scale_color_identity() +  # Use the actual color values
  stat_summary(fun.data = "mean_sdl", fun.args = list(mult = 1), 
               geom = "pointrange", color = "black", size = 1.75, alpha = 1.0) +
  theme_minimal() +
  labs(title = "All data oN mean PhyloP", x = "Node category SVM", y = "oN mean PhyloP") +
geom_boxplot(outlier.shape = NA, alpha=0.01, size=1.25)

print(Nodecat_on_meanphylop_meplot_svm)
```

```
ggsave("Nodecat_on_meanphylop_meplot_svm_combined_color.pdf", plot = Nodecat_on_meanphylop_meplot_svm, width = 5, height = 10)
```

We see that the distribution of constraint values matches the
hypothesis.

## Declassification of Uncertain identity nodes

However, there may be some nodes that are classified, but don’t have
strong support for either class. We have a closer look at the output of
the SVM based classification through looking at the decision.values
object. Here and elsewhere, we always count the rows of the dataset to
keep track of what’s included. Loosely speaking, decision values of
>+1 and <-1 (representing the hyperplane dividing the categories),
are considered as well supported, but there is no exact cut-off point.
Here we assume that values closer to zero than +0.7 and -0.7 should not
be classified as H,I or P but instead as “Uncertain”. We go with the
same procedure as the SVM and do a majority vote (if at least 2 out of
the three decision vectors fall below the threshold the node is
classified as uncertain).

```
# make a new test_set not to disturb the other one we just made
test_set2 <- dataset[is.na(dataset$DFA_Nodecat), ]
print("row count of test_set")
```

```
## [1] "row count of test_set"
```

```
print(nrow(test_set2))
```

```
## [1] 13397
```

```
svm_predictions2 <- predict(svm_model, newdata = test_set_for_prediction, decision.values = TRUE)
svm_predictions_vector <- as.vector(svm_predictions2)
decision_values <- attr(svm_predictions2, "decision.values")

print(str(decision_values))
```

```
##  num [1:13397, 1:3] 1.64 -1.48 -3.99 -2.73 -3.57 ...
##  - attr(*, "dimnames")=List of 2
##   ..$ : chr [1:13397] "1" "2" "3" "4" ...
##   ..$ : chr [1:3] "H/I" "H/P" "I/P"
## NULL
```

```
if (is.matrix(decision_values) || is.data.frame(decision_values)) {
    colnames(decision_values) <- gsub("/", "_", colnames(decision_values))
    print(colnames(decision_values))
} else {
    print("decision_values is not a matrix or data frame.")
}
```

```
## [1] "H_I" "H_P" "I_P"
```

```
decision_values <- as.data.frame(decision_values)


# Attach predictions and decision values back to the test_set
test_set2$Predicted_Class3 <- svm_predictions_vector
test_set2$decision_values_HI <- NA #housekeeping just in case
test_set2$decision_values_HP <- NA
test_set2$decision_values_IP <- NA

test_set2$decision_values_HI <- decision_values$H_I
test_set2$decision_values_HP <- decision_values$H_P
test_set2$decision_values_IP <- decision_values$I_P

test_set2$decision_values_HI <- as.numeric(test_set2$decision_values_HI)
test_set2$decision_values_HP <- as.numeric(test_set2$decision_values_HP)
test_set2$decision_values_IP <- as.numeric(test_set2$decision_values_IP)

# Perform the reclassification of weakly supported nodes as Uncertain.
test_set2$Predicted_Class4 <- test_set2$Predicted_Class3
confidence_threshold <- 0.7
for (i in 1:nrow(test_set2)) {
    values <- c(test_set2[i, "decision_values_HI"], 
                test_set2[i, "decision_values_HP"], 
                test_set2[i, "decision_values_IP"])
    values_below_threshold <- sum(abs(values) < confidence_threshold)
    if (values_below_threshold >= 2) {
        test_set2$Predicted_Class4[i] <- 'Uncertain'
    }
}
head(test_set2$Predicted_Class4)
```

```
## [1] "H" "I" "I" "I" "I" "I"
```

```
print("row count of test set2 still missing the original training data")
```

```
## [1] "row count of test set2 still missing the original training data"
```

```
print(nrow(test_set2))
```

```
## [1] 13397
```

```
table(test_set2$Predicted_Class4)
```

```
## 
##         H         I         P Uncertain 
##       136      9358      2856      1047
```

```
# Add training data back in and merge subsets
initial_classified_copy$Predicted_Class4 <- NA
initial_classified_copy$decision_values_HI <- NA
initial_classified_copy$decision_values_HP <- NA
initial_classified_copy$decision_values_IP <- NA
test_set2$DFA_Nodecat <- NULL
test_set2$DFA_Nodecat <- NA
test_set2$Predicted_Class4 <- as.factor(test_set2$Predicted_Class4)
common_cols <- intersect(names(test_set2), names(initial_classified_copy))
test_set2_common <- test_set2[, common_cols]
initial_classified_copy_common <- initial_classified_copy[, common_cols]
test_set3 <- rbind(test_set2_common, initial_classified_copy_common)
test_set3$Predicted_Class4[is.na(test_set3$Predicted_Class4)] <- test_set3$DFA_Nodecat[is.na(test_set3$Predicted_Class4)]

print("row count of test set2 after adding the original training data")
```

```
## [1] "row count of test set2 after adding the original training data"
```

```
print(nrow(test_set3))
```

```
## [1] 16957
```

```
table(test_set3$Predicted_Class4)
```

```
## 
##         H         I         P Uncertain 
##       306     11052      4552      1047
```

Plot the decision values and the new distribution of mean Phylop
after declassing the Uncertain nodes.

```
test_set3$Predicted_Class4 <- factor(test_set3$Predicted_Class4)
num_classes <- length(unique(test_set3$Predicted_Class4))
viridis_colors <- viridis::viridis(num_classes)
color_mapping <- setNames(viridis_colors, unique(test_set3$Predicted_Class4))
fig2 <- plot_ly(data = test_set3, 
               x = ~decision_values_HI, 
               y = ~decision_values_HP, 
               z = ~decision_values_IP, 
               type = 'scatter3d', 
               mode = 'markers',
               marker = list(size = 5, 
                             color = ~color_mapping[Predicted_Class4], 
                             showscale = FALSE, opacity=0.5))  # Remove colorbar
fig2 <- fig2 %>% layout(title = "3D Scatter Plot of Decision Values",
                      scene = list(xaxis = list(title = 'Decision Value H/I'),
                                   yaxis = list(title = 'Decision Value H/P'),
                                   zaxis = list(title = 'Decision Value I/P')))
fig2
```

```
#just redoing the order Norm transformation
"orderNorm_meanPhylop" %in% names(test_set3)
```

```
## [1] FALSE
```

```
test_set3$orderNorm_meanPhylop <- NULL
"orderNorm_meanPhylop" %in% names(test_set3)
```

```
## [1] FALSE
```

```
ON_meanPhylop <- orderNorm(test_set3$meanPhylop)
orderNorm_meanPhylop <- predict(ON_meanPhylop)
test_set3 <- cbind(test_set3, orderNorm_meanPhylop)
"orderNorm_meanPhylop" %in% names(test_set3)
```

```
## [1] TRUE
```

```
plot_data2 <- test_set3

lower_quantile <- quantile(plot_data2$orderNorm_meanPhylop, probs = 0.05, na.rm = TRUE)
upper_quantile <- quantile(plot_data2$orderNorm_meanPhylop, probs = 0.95, na.rm = TRUE)

assign_combined_color <- function(value, lower_quantile, upper_quantile, lower_color, upper_color, middle_color_scale) {
  if (is.na(value)) {
    return(NA)  # Keep NA if the value is NA
  } else if (value <= lower_quantile) {
    return(lower_color)
  } else if (value >= upper_quantile) {
    return(upper_color)
  } else {
    normalized_value <- (value - lower_quantile) / (upper_quantile - lower_quantile)
    return(middle_color_scale(normalized_value))
  }
}

middle_color_scale <- scales::col_numeric(palette = viridis_pal(option = "cividis")(100), domain = c(0, 1))

plot_data2$combined_color <- mapply(assign_combined_color,
                                        plot_data2$orderNorm_meanPhylop,
                                        MoreArgs = list(
                                          lower_quantile = lower_quantile,
                                          upper_quantile = upper_quantile,
                                          lower_color = "#002051",
                                          upper_color = "#fdea45",
                                          middle_color_scale = middle_color_scale
                                        ))

Nodecat_on_meanphylop_meplot_uncertain_svm <- ggplot(plot_data2, aes(x = Predicted_Class4, y = orderNorm_meanPhylop)) +
  geom_jitter(aes(colour = combined_color), alpha = 0.3, shape = 16, size=4) +
  scale_color_identity() +  # Use the actual color values
  stat_summary(fun.data = "mean_sdl", fun.args = list(mult = 1), 
               geom = "pointrange", color = "black", size = 1.75, alpha = 1.0) +
  theme_minimal() +
                 scale_x_discrete(guide = guide_axis(n.dodge = 2)) +
  labs(title = "Uncertain oN mean Phylop", x = "Node category SVM", y = "oN mean Phylop") +
geom_boxplot(outlier.shape = NA, alpha=0.01, size=1.25)

print(Nodecat_on_meanphylop_meplot_uncertain_svm)
```

```
ggsave("Nodecat_on_meanphylop_meplot_svm_uncertain.pdf", plot = Nodecat_on_meanphylop_meplot_uncertain_svm, width = 5, height = 10)

write.csv(test_set3, "mammals_stats_predicted_Classes.csv", row.names=FALSE)
```

remove the uncertain nodes from the following analysis

```
test_set4 <- test_set3[test_set3$Predicted_Class4 != "Uncertain", ]
test_set4$Predicted_Class4 <- droplevels(test_set4$Predicted_Class4)
print("row count of test set4 with uncertain node rows removed")
```

```
## [1] "row count of test set4 with uncertain node rows removed"
```

```
print(nrow(test_set4))
```

```
## [1] 15910
```

```
table(test_set4$Predicted_Class4)
```

```
## 
##     H     I     P 
##   306 11052  4552
```

Perform ANOVA to test for effect of node class on orderNorm
transformed mean Phylop

```
anova_result <- aov(orderNorm_meanPhylop ~ Predicted_Class4, data = test_set4)
summary(anova_result)
```

```
##                     Df Sum Sq Mean Sq F value Pr(>F)    
## Predicted_Class4     2    428  213.77   218.6 <2e-16 ***
## Residuals        14825  14500    0.98                   
## ---
## Signif. codes:  0 '***' 0.001 '**' 0.01 '*' 0.05 '.' 0.1 ' ' 1
## 1082 observations deleted due to missingness
```

Node class significantly predicts oN meanPhylop

Perform post-hoc Emmeans test

```
anova_result2 <- aov(orderNorm_meanPhylop ~ Predicted_Class4, data = test_set4)
anova_posthoc_pairs <- summary(emmeans(anova_result2, 
                pairwise ~ Predicted_Class4, adjust="fdr")$contrast) %>% 
  as.data.frame()
anova_posthoc_pairs %>% 
  kbl(digits = 4, 
      caption = "Pairwise tests of oN_meanPhylop by node cateogry FDR adjustment") %>% 
  kable_classic(full_width = F, html_font = "Cambria")
```

Pairwise tests of oN\_meanPhylop by node cateogry FDR adjustment

| contrast | estimate | SE | df | t.ratio | p.value |
| --- | --- | --- | --- | --- | --- |
| H - I | 0.5052 | 0.0582 | 14825 | 8.6813 | 0 |
| H - P | 0.8364 | 0.0594 | 14825 | 14.0751 | 0 |
| I - P | 0.3312 | 0.0182 | 14825 | 18.1773 | 0 |

Since the p-values could be influenced by the large sample sizes, we
add eta-squared for effect of the whole model, including the Evidence
interpretations of eta-squared by Cohen 1992.

## Result of ANOVA and effect sizes

```
anova_result <- car::Anova(aov(orderNorm_meanPhylop ~ Predicted_Class4, data = test_set4), type = 2)
anova_result_df <- tidy(anova_result) %>%
  as.data.frame()

# Table summary


anova_result_df <- sjstats::anova_stats(car::Anova(aov(orderNorm_meanPhylop ~ Predicted_Class4, data = test_set4), type = 2)) %>%
  dplyr::select(-c(etasq, omegasq, epsilonsq, power, cohens.f))


# Effect size
anova_result_df$eta2.int=interpret_eta_squared(
                              anova_result_df$partial.etasq,
                              rules="cohen1992")

anova_result_df <- anova_result_df %>% 
    dplyr::rename("P"="p.value")  %>% 
  mutate("Significance" = case_when(
    P >= 0.05 ~ "",
    P >= 0.01 ~ "*",
    P >= 0.001 ~ "**",
    P >= 0.0001 ~ "***",
    P < 0.0001 ~ "****",
    TRUE ~ NA
  )) %>%
  mutate("Evidence" = case_when(
    P >= 0.1 ~ "Little or none",
    P >= 0.05 ~ "Weak",
    P >= 0.01 ~ "Moderate",
    P >= 0.001 ~ "Strong",
    P < 0.0001 ~ "Very strong",
    TRUE ~ NA
  )) 

anova_result_df %>% 
  kbl(digits = 4,
  caption = "Analysis of variance for orderNorm meanPhylop - full data") %>%
  kable_classic(full_width = F, html_font = "Cambria")
```

Analysis of variance for orderNorm meanPhylop - full data

|  | term | sumsq | meansq | df | statistic | P | partial.etasq | partial.omegasq | eta2.int | Significance | Evidence |
| --- | --- | --- | --- | --- | --- | --- | --- | --- | --- | --- | --- |
| Predicted\_Class4 | Predicted\_Class4 | 427.544 | 213.772 | 2 | 218.566 | 0 | 0.029 | 0.029 | small | \*\*\*\* | Very strong |
| …2 | Residuals | 14499.801 | 0.978 | 14825 | NA | NA | NA | NA | NA | NA | NA |

The effect is small but significant and evidence is very strong.

Since the p-values could be influenced by the large sample sizes, we
add Cohens D for effect size of pairwise comparisons, and using the
Evidence interpretations of Cohen’s D amd Eta squared as mentioned in
Sawilovsky, 2009.

```
# Effect size
anova_posthoc_pairs4 <- anova_posthoc_pairs %>% 
    dplyr::rename("P"="p.value")  %>% 
  mutate("Significance" = case_when(
    P >= 0.05 ~ "",
    P >= 0.01 ~ "*",
    P >= 0.001 ~ "**",
    P >= 0.0001 ~ "***",
    P < 0.0001 ~ "****",
    TRUE ~ NA
  )) %>%
  mutate("Evidence" = case_when(
    P >= 0.1 ~ "Little or none",
    P >= 0.05 ~ "Weak",
    P >= 0.01 ~ "Moderate",
    P >= 0.001 ~ "Strong",
    P < 0.0001 ~ "Very strong",
    TRUE ~ NA
  )) 

anova_posthoc_pairs4$d <- t_to_d(t = anova_posthoc_pairs4$t.ratio, 
                  df = 103)[1]$d
anova_posthoc_pairs4$r <- t_to_r(t = anova_posthoc_pairs4$t.ratio, 
                  df = 103)[1]$r
anova_posthoc_pairs4$O2 <- t_to_omega2(t = anova_posthoc_pairs4$t.ratio, 
                  df = 103)[1]$'Omega2 (partial)' 
anova_posthoc_pairs4$eta2 <- t_to_eta2(t = anova_posthoc_pairs4$t.ratio, 
                      df = 103)[1]$Eta2_partial
 
anova_posthoc_pairs4$d.int=interpret_cohens_d(anova_posthoc_pairs4$d,
                             rules="sawilowsky2009")
anova_posthoc_pairs4$eta2.int=interpret_eta_squared(anova_posthoc_pairs4$eta2,
                              rules="cohen1992")

anova_posthoc_pairs4 %>%
  kbl(digits = 4,caption = "Pairwise effect sizes for Nodecat"  ) %>%
  kable_classic(full_width = F, html_font = "Cambria")
```

Pairwise effect sizes for Nodecat

| contrast | estimate | SE | df | t.ratio | P | Significance | Evidence | d | r | eta2 | d.int | eta2.int |
| --- | --- | --- | --- | --- | --- | --- | --- | --- | --- | --- | --- | --- |
| H - I | 0.5052 | 0.0582 | 14825 | 8.6813 | 0 | \*\*\*\* | Very strong | 1.7108 | 0.6500 | 0.4225 | very large | large |
| H - P | 0.8364 | 0.0594 | 14825 | 14.0751 | 0 | \*\*\*\* | Very strong | 2.7737 | 0.8111 | 0.6579 | huge | large |
| I - P | 0.3312 | 0.0182 | 14825 | 18.1773 | 0 | \*\*\*\* | Very strong | 3.5821 | 0.8731 | 0.7624 | huge | large |

The node categories all have significant pairwise differences in
constraint with a very large to huge effect size.

# Test of Hypothesis 3. Hibernation-relevant genes are located in intermediate nodes and differ in constraint values

Here we first remove the genes which aren’t relevant for
Hibernation.

```
test_set4$Direction <- as.factor(test_set4$Direction)
table(test_set4$Direction)
```

```
## 
##                   1 slower 2 faster 
##    12997     2896        8        9
```

```
rows_with_empty_level <- which(test_set4$Direction == "")
rm_Dir <- test_set4[-rows_with_empty_level, ]
levels(rm_Dir$Direction)
```

```
## [1] ""         " "        "1 slower" "2 faster"
```

```
rm_Dir$Direction[rm_Dir$Direction == ""] <- NA
rm_Dir$Direction[rm_Dir$Direction == " "] <- NA
rm_Dir$Direction <- droplevels(rm_Dir$Direction)
rm_Dir <- rm_Dir[!is.na(rm_Dir$Direction), ]
levels(rm_Dir$Direction)
```

```
## [1] "1 slower" "2 faster"
```

```
table(rm_Dir$Predicted_Class4)
```

```
## 
##  H  I  P 
##  1 12  4
```

The majority of the hibernation genes are in the I node category as
expected

```
Direction_oN_mean_phylop_box <- ggplot(rm_Dir, aes(x = Direction, y = orderNorm_meanPhylop)) +
  theme_minimal() +
  geom_jitter(aes(shape = Predicted_Class4, color = Predicted_Class4), alpha = 0.6, size = 6, width=0.2) +
  scale_color_viridis_d() +
  geom_boxplot(aes(fill = Direction), outlier.shape = NA, alpha = 0.3) +
  labs(title = "All data oN mean Phylop", x = "Direction", y = "oN mean PhyloP") +
  scale_fill_viridis_d() +
  scale_shape_manual(values = c(16, 17, 15))  # Closed shapes
print(Direction_oN_mean_phylop_box)
```

```
ggsave("Direction_oN_mean_phylop_box.pdf", Direction_oN_mean_phylop_box, width = 5, height = 10)
```

Genes accelerating evolutionary rate in aligned with the emergence of
hibernation are more constrained than genes lowering their evolutionary
rate in aligned with the emergene of hibernation

## Effect of Node category and direction of rate change rho on Hibernation-relevant genes

Fit a linear model for the effect of Node category and direction of
rate change rho on Hibernation-relevant genes, followed by post hoc
tests and effect size estimation as described above.

```
anova_result <- car::Anova(aov(orderNorm_meanPhylop ~ Direction + Predicted_Class4, data = rm_Dir), type = 2)
anova_result_df <- tidy(anova_result) %>%
  as.data.frame()
anova_result_df <- sjstats::anova_stats(car::Anova(aov(orderNorm_meanPhylop ~ Direction + Predicted_Class4, data = rm_Dir), type = 2)) %>% 
  dplyr::select(-c(etasq, omegasq, epsilonsq, power, cohens.f))


anova_result_df$eta2.int=interpret_eta_squared(
                              anova_result_df$partial.etasq,
                              rules="cohen1992")

anova_result_df <- anova_result_df %>% 
    dplyr::rename("P"="p.value")  %>% 
  mutate("Significance" = case_when(
    P >= 0.05 ~ "",
    P >= 0.01 ~ "*",
    P >= 0.001 ~ "**",
    P >= 0.0001 ~ "***",
    P < 0.0001 ~ "****",
    TRUE ~ NA
  )) %>%
  mutate("Evidence" = case_when(
    P >= 0.1 ~ "Little or none",
    P >= 0.05 ~ "Weak",
    P >= 0.01 ~ "Moderate",
    P >= 0.001 ~ "Strong",
    P < 0.0001 ~ "Very strong",
    TRUE ~ NA
  )) 

anova_result_df %>% 
  kbl(digits = 4,
  caption = "Analysis of variance for orderNorm meanPhylop by Direction - full data") %>%
  kable_classic(full_width = F, html_font = "Cambria")
```

Analysis of variance for orderNorm meanPhylop by Direction - full data

|  | term | sumsq | meansq | df | statistic | P | partial.etasq | partial.omegasq | eta2.int | Significance | Evidence |
| --- | --- | --- | --- | --- | --- | --- | --- | --- | --- | --- | --- |
| Direction | Direction | 5.163 | 5.163 | 1 | 6.229 | 0.027 | 0.324 | 0.235 | large |  | Moderate |
| Predicted\_Class4 | Predicted\_Class4 | 1.545 | 0.773 | 2 | 0.932 | 0.419 | 0.125 | -0.008 | small |  | Little or none |
| …3 | Residuals | 10.776 | 0.829 | 13 | NA | NA | NA | NA | NA | NA | NA |

Since the p-values could be influenced by the large sample sizes, we
add Cohens D for effect size of pairwise comparisons, and using the
Evidence interpretations of Cohen’s D amd Eta squared as mentioned in
Sawilovsky, 2009.

```
# Effect size
anova_posthoc_pairs2 <- anova_posthoc_pairs %>% 
    dplyr::rename("P"="p.value")  %>% 
  mutate("Significance" = case_when(
    P >= 0.05 ~ "",
    P >= 0.01 ~ "*",
    P >= 0.001 ~ "**",
    P >= 0.0001 ~ "***",
    P < 0.0001 ~ "****",
    TRUE ~ NA
  )) %>%
  mutate("Evidence" = case_when(
    P >= 0.1 ~ "Little or none",
    P >= 0.05 ~ "Weak",
    P >= 0.01 ~ "Moderate",
    P >= 0.001 ~ "Strong",
    P < 0.0001 ~ "Very strong",
    TRUE ~ NA
  )) 

anova_posthoc_pairs2$d <- t_to_d(t = anova_posthoc_pairs2$t.ratio, 
                  df = 103)[1]$d
anova_posthoc_pairs2$r <- t_to_r(t = anova_posthoc_pairs2$t.ratio, 
                  df = 103)[1]$r
anova_posthoc_pairs2$O2 <- t_to_omega2(t = anova_posthoc_pairs2$t.ratio, 
                  df = 103)[1]$'Omega2 (partial)' 
anova_posthoc_pairs2$eta2 <- t_to_eta2(t = anova_posthoc_pairs2$t.ratio, 
                      df = 103)[1]$Eta2_partial
 
anova_posthoc_pairs2$d.int=interpret_cohens_d(anova_posthoc_pairs2$d,
                             rules="sawilowsky2009")
anova_posthoc_pairs2$eta2.int=interpret_eta_squared(anova_posthoc_pairs2$eta2,
                              rules="cohen1992")

anova_posthoc_pairs2 %>%
  kbl(digits = 4,caption = "Pairwise effect sizes for Direction"  ) %>%
  kable_classic(full_width = F, html_font = "Cambria")
```

Pairwise effect sizes for Direction

| contrast | estimate | SE | df | t.ratio | P | Significance | Evidence | d | r | eta2 | d.int | eta2.int |
| --- | --- | --- | --- | --- | --- | --- | --- | --- | --- | --- | --- | --- |
| H - I | 0.5052 | 0.0582 | 14825 | 8.6813 | 0 | \*\*\*\* | Very strong | 1.7108 | 0.6500 | 0.4225 | very large | large |
| H - P | 0.8364 | 0.0594 | 14825 | 14.0751 | 0 | \*\*\*\* | Very strong | 2.7737 | 0.8111 | 0.6579 | huge | large |
| I - P | 0.3312 | 0.0182 | 14825 | 18.1773 | 0 | \*\*\*\* | Very strong | 3.5821 | 0.8731 | 0.7624 | huge | large |

# Test of Hypothesis 4. The intermediate position of hibernation-relevant genes in the network is not just an outcome of chance due to predominance of this node category within the interactome.

We can compare the distribution of ASPL, NC, BC, and DFA\_nodecat of
1000 iterations of 18 randomly picked genes, and the actual values of
these genes. Differences in distributions are assessed with
Kolmogorov-Smirnov tests.

## Tests for Average Shortest Path Length

```
set.seed(123)
table(test_set3$Direction)
```

```
## 
##                   1 slower 2 faster 
##    13850     3089        8       10
```

```
filtered_data3 <- test_set3 %>%
 filter(Direction %in% c("1 slower", "2 faster"))

num_samples <- 1000
sample_size <- 18

sample_means <- replicate(num_samples, {
  sample_rows <- sample(1:nrow(test_set3), size = sample_size)
  sampled_data <- test_set3$AverageShortestPathLength[sample_rows]
  mean(sampled_data)
}, simplify = "data.frame")

sample_means_df <- data.frame(AverageShortestPathLength = sample_means)
ks_test_1slower_vs_sample_means <- ks.test(filtered_data3$AverageShortestPathLength[filtered_data3$Direction == "1 slower"], sample_means_df$AverageShortestPathLength)

ks_test_2faster_vs_sample_means <- ks.test(filtered_data3$AverageShortestPathLength[filtered_data3$Direction == "2 faster"],sample_means_df$AverageShortestPathLength)

ks_test_hib_vs_sample_means <-ks.test(filtered_data3$AverageShortestPathLength, sample_means_df$AverageShortestPathLength)


ks_test_results <- data.frame(
  Test = c("1 slower vs. simulated means", "2 faster vs. simulated means", "hibernationgenes vs. simulated means"),
  Statistic = c(ks_test_1slower_vs_sample_means$statistic, 
                ks_test_2faster_vs_sample_means$statistic, 
                ks_test_hib_vs_sample_means$statistic),
  P_Value = c(ks_test_1slower_vs_sample_means$p.value, 
              ks_test_2faster_vs_sample_means$p.value, 
              ks_test_hib_vs_sample_means$p.value)
)
kable(ks_test_results, caption = "Kolmogorov-Smirnov Test Results for ASPL")
```

Kolmogorov-Smirnov Test Results for ASPL

| Test | Statistic | P\_Value |
| --- | --- | --- |
| 1 slower vs. simulated means | 0.3740000 | 0.1675508 |
| 2 faster vs. simulated means | 0.3000000 | 0.3349494 |
| hibernationgenes vs. simulated means | 0.3323333 | 0.0402521 |

## Tests for Betweenness Centrality

```
set.seed(123)
num_samples <- 1000
sample_size <- 18

sample_means <- replicate(num_samples, {
  sample_rows <- sample(1:nrow(test_set3), size = sample_size)
  sampled_data <- test_set3$BetweennessCentrality[sample_rows]
  mean(sampled_data)
}, simplify = "data.frame")
sample_means_df <- data.frame(BetweennessCentrality = sample_means)

ks_test_1slower_vs_sample_means <- ks.test(filtered_data3$BetweennessCentrality[filtered_data3$Direction == "1 slower"], 
                                            sample_means_df$BetweennessCentrality)
ks_test_2faster_vs_sample_means <- ks.test(filtered_data3$BetweennessCentrality[filtered_data3$Direction == "2 faster"], 
                                            sample_means_df$BetweennessCentrality)
ks_test_hib_vs_sample_means <- ks.test(filtered_data3$BetweennessCentrality, 
                                            sample_means_df$BetweennessCentrality)

ks_test_results <- data.frame(
  Test = c("1 slower vs. simulated means", "2 faster vs. simulated means", "hibernationgenes vs. simulated means"),
  Statistic = c(ks_test_1slower_vs_sample_means$statistic, 
                ks_test_2faster_vs_sample_means$statistic, 
                ks_test_hib_vs_sample_means$statistic),
  P_Value = c(ks_test_1slower_vs_sample_means$p.value, 
              ks_test_2faster_vs_sample_means$p.value, 
              ks_test_hib_vs_sample_means$p.value)
)
kable(ks_test_results, caption = "Kolmogorov-Smirnov Test Results for BC")
```

Kolmogorov-Smirnov Test Results for BC

| Test | Statistic | P\_Value |
| --- | --- | --- |
| 1 slower vs. simulated means | 0.5000000 | 0.0235732 |
| 2 faster vs. simulated means | 0.5010000 | 0.0138819 |
| hibernationgenes vs. simulated means | 0.4565556 | 0.0012581 |

## Tests for Neighborhood Connectivity

```
set.seed(123)
sample_means <- replicate(num_samples, {
  sample_rows <- sample(1:nrow(test_set3), size = sample_size)
  sampled_data <- test_set3$NeighborhoodConnectivity[sample_rows]
  mean(sampled_data)
}, simplify = "data.frame")
sample_means_df <- data.frame(NeighborhoodConnectivity = sample_means)


ks_test_1slower_vs_sample_means <- ks.test(filtered_data3$NeighborhoodConnectivity[filtered_data3$Direction == "1 slower"], 
                                            sample_means_df$NeighborhoodConnectivity)
ks_test_2faster_vs_sample_means <- ks.test(filtered_data3$NeighborhoodConnectivity[filtered_data3$Direction == "2 faster"], 
                                            sample_means_df$NeighborhoodConnectivity)
ks_test_hib_vs_sample_means <- ks.test(filtered_data3$NeighborhoodConnectivity, 
                                            sample_means_df$NeighborhoodConnectivity)

ks_test_results <- data.frame(
  Test = c("1 slower vs. simulated means", "2 faster vs. simulated means", "hibernationgenes vs. simulated means"),
  Statistic = c(ks_test_1slower_vs_sample_means$statistic, 
                ks_test_2faster_vs_sample_means$statistic, 
                ks_test_hib_vs_sample_means$statistic),
  P_Value = c(ks_test_1slower_vs_sample_means$p.value, 
              ks_test_2faster_vs_sample_means$p.value, 
              ks_test_hib_vs_sample_means$p.value)
)
kable(ks_test_results, caption = "Kolmogorov-Smirnov Test Results for NC")
```

Kolmogorov-Smirnov Test Results for NC

| Test | Statistic | P\_Value |
| --- | --- | --- |
| 1 slower vs. simulated means | 0.382 | 0.1508962 |
| 2 faster vs. simulated means | 0.654 | 0.0004195 |
| hibernationgenes vs. simulated means | 0.499 | 0.0002998 |

## Test for meanPhylop

```
set.seed(123)
sample_means <- replicate(num_samples, {
  sample_rows <- sample(1:nrow(test_set3), size = sample_size)
  sampled_data <- test_set3$meanPhylop[sample_rows]
  mean(sampled_data)
}, simplify = "data.frame")
sample_means_df <- data.frame(meanPhylop = sample_means)


ks_test_1slower_vs_sample_means <- ks.test(filtered_data3$meanPhylop[filtered_data3$Direction == "1 slower"], 
                                            sample_means_df$meanPhylop)
ks_test_2faster_vs_sample_means <- ks.test(filtered_data3$meanPhylop[filtered_data3$Direction == "2 faster"], 
                                            sample_means_df$meanPhylop)
ks_test_hib_vs_sample_means <- ks.test(filtered_data3$meanPhylop, 
                                            sample_means_df$meanPhylop)
ks_test_results <- data.frame(
  Test = c("1 slower vs. simulated means", "2 faster vs. simulated means", "hibernationgenes vs. simulated means"),
  Statistic = c(ks_test_1slower_vs_sample_means$statistic, 
                ks_test_2faster_vs_sample_means$statistic, 
                ks_test_hib_vs_sample_means$statistic),
  P_Value = c(ks_test_1slower_vs_sample_means$p.value, 
              ks_test_2faster_vs_sample_means$p.value, 
              ks_test_hib_vs_sample_means$p.value)
)
kable(ks_test_results, caption = "Kolmogorov-Smirnov Test Results for meanPhylop")
```

Kolmogorov-Smirnov Test Results for meanPhylop

| Test | Statistic | P\_Value |
| --- | --- | --- |
| 1 slower vs. simulated means | 0.3636364 | 0.2028744 |
| 2 faster vs. simulated means | 0.7848485 | 0.0000009 |
| hibernationgenes vs. simulated means | 0.5808081 | 0.0000069 |
